# Rikkunshito prevents paclitaxel-induced peripheral neuropathy through the suppression of the nuclear factor kappa B (NFκB) phosphorylation in spinal cord of mice

**DOI:** 10.1371/journal.pone.0171819

**Published:** 2017-02-09

**Authors:** Junzo Kamei, Shunsuke Hayashi, Akane Sakai, Yuki Nakanishi, Misa Kai, Megumi Ikegami, Hiroko Ikeda

**Affiliations:** Department of Pathophysiology and Therapeutics, Hoshi University School of Pharmacy and Pharmaceutical Sciences, Shinagawa-ku, Tokyo, Japan; University of Sao Paulo, BRAZIL

## Abstract

Peripheral neuropathy is the major side effect caused by paclitaxel, a microtubule-binding antineoplastic drug. Paclitaxel-induced peripheral neuropathy causes a long-term negative impact on the patient's quality of life. However, the mechanism underlying paclitaxel-induced peripheral neuropathy is still unknown, and there is no established treatment. Ghrelin is known to attenuate thermal hyperalgesia and mechanical allodynia in chronic constriction injury of the sciatic nerve, and inhibit the activation of nuclear factor kappa B (NFκB) in the spinal dorsal horn. Rikkunshito (RKT), a kampo medicine, increases the secretion of ghrelin in rodents and humans. Thus, RKT may attenuate paclitaxel-induced peripheral neuropathy by inhibiting phosphorylated NFκB (pNFκB) in the spinal cord. We found that paclitaxel dose-dependently induced mechanical hyperalgesia in mice. Paclitaxel increased the protein levels of spinal pNFκB, but not those of spinal NFκB. NFκB inhibitor attenuated paclitaxel-induced mechanical hyperalgesia suggesting that the activation of NFκB mediates paclitaxel-induced hyperalgesia. RKT dose-dependently attenuated paclitaxel-induced mechanical hyperalgesia. Ghrelin receptor antagonist reversed the RKT-induced attenuation of paclitaxel-induced mechanical hyperalgesia. RKT inhibited the paclitaxel-induced increase in the protein levels of spinal pNFκB. Taken together, the present study indicates that RKT exerts an antihyperalgesic effect in paclitaxel-induced neuropathic pain by suppressing the activation of spinal NFκB.

## Introduction

Peripheral neuropathy is a common adverse effect of anti-cancer drugs, including the vinca alkaloid vincristine, the taxane paclitaxel, and the platinum-based drug oxaliplatin. Chemotherapy-induced peripheral neuropathy (CIPN) can be very painful [1]. CIPN is characterized by sensory loss, paresthesia, dysesthesia, numbness, and tingling, often aggravated by neuropathic pain. Presently, the treatment options for CIPN are quite limited. Opioids are used for the treatment of cancer pain, but are not suitable for chronic pain. However, there are new groups of drugs for the treatment of neuropathic pain, such as topical agents, tricyclic antidepressants, serotonin noradrenaline reuptake inhibitors (duloxetine and venlafaxine), gabapentin, pregabalin, corticosteroids, bisphosphonates, N-methyl-D-aspartate antagonists, and cannabinoids [2].

Paclitaxel is a microtubule-binding antineoplastic drug. It is an effective chemotherapeutic agent that is broadly used for the treatment of breast, ovarian, and non-small-cell lung cancer [3]. Peripheral neuropathy is one of its major side effects. Paclitaxel-induced peripheral neuropathy is characterized by a sensory abnormality in the extremities that usually occurs in a stocking-and-glove distribution together with motor dysfunction in patients [4]. Paclitaxel-induced peripheral neuropathy may persist for months to years [5], and thus can have a long-term negative impact on the patient's quality of life. However, the mechanisms that underlie paclitaxel-induced peripheral neuropathy are still unknown, and there is no established treatment.

NFκB has been suggested to be involved in chronic neuropathic pain. It was reported that the percentages of activated NFκB immunoreactive neurons in the side of the spinal cord ipsilateral to partial sciatic nerve injury in rats were significantly increased [6]. Several reports have shown that NFκB pathway inhibitors, such as pyrrolidine dithiocarbamate and S1627 (inhibitory kappa B (IκB) kinase (IKK) inhibitor), attenuate chronic pain [7–9]. However, there is no report that whether NFκB pathway is involved in paclitaxel-induced peripheral neuropathy.

Rikkunshito (RKT; TJ-43), which is a Kampo (Japanese herbal) medicine, is known as a prokinetic agent for patients with several diseases such as gastro-esophageal reflux disease (GERD), non-erosive reflux disease (NERD), and functional dyspepsia [10–15]. It has been reported that oral administration of RKT increases the secretion of ghrelin in rodents and humans [16–18]. Ghrelin, the endogenous ligand for growth hormone secretagogue receptor 1a (GHSR-1a), has been shown to prevent the release of proinflammatory cytokines. Intrathecal injection of ghrelin clearly attenuated thermal hyperalgesia and mechanical allodynia in chronic constriction injury (CCI) of the sciatic nerve and reduced the activation of nuclear factor kappa B (NFκB) p65 in the spinal dorsal horn [19]. However, it has no reported that whether RKT attenuates paclitaxel-induced peripheral neuropathy.

Taken together, these results suggest that RKT may attenuate paclitaxel-induced peripheral neuropathy via the inhibition of phosphorylated NFκB (pNFκB) in the spinal cord. The present study, we examined the effects of NFκB in paclitaxel-induced neuropathic pain. In addition, we also investigated the effects of RKT in paclitaxel-induced neuropathic pain.

## Materials and methods

### Ethics statement

The animal protocols were approved as conforming to the Guide for the Care and Use of Laboratory Animals by the issuing committee (Committee on the Care and Use of Laboratory Animals of Hoshi University (Permit Number: 11–101), which is accredited by the Ministry of Education, Culture, Sports, Science, and Technology, Japan). All surgery was performed under sodium pentobarbital anesthesia, and all efforts were made to minimize suffering.

### Animals

Male ICR 6-week-old mice (Tokyo Animal Laboratories Inc., Tokyo, Japan), weighing 20 to 30 g, were used in this study. They had free access to food and water in an animal room that was maintained at 24±1°C with a 12-h light-dark cycle (light on at 08:00, light off at 20:00).

### Drugs

Paclitaxel (LKT Laboratories inc, St. Paul, MN, USA) was dissolved in 10% dimethyl sulfoxide (10%DMSO). Ammonium pyrrolidine dithiocarbamate (PDTC; Sigma-Aldrich, St Louis, MO, USA), an NFκB inhibitor, and [D-Lys^3^]-GHRP-6 (Tocris, Bristol, United Kingdom), a ghrelin receptor antagonist, were dissolved in saline. RKT (Tsumura, Tokyo, Japan) was dissolved in distilled water (DW).

Paclitaxel was injected intraperitoneally (i.p.) at a dose of 2 or 5 mg/kg. PDTC was injected intrathecally (i.t.) at a dose of 100 ng/5μL. [D-Lys^3^]-GHRP-6 was injected i.p. at a dose of 10 mg/kg. RKT was administered orally (p.o.) at a dose of 0.1, 0.3, or 1 g/kg. Control animals were injected with the respective vehicle for each drug.

### Paclitaxel-induced neuropathy model and experiment schedule

This study was comprised of 8 experiments, and mechanical nociceptive thresholds were measured once a day in all experiments.

In experiment 1, animals were treated with a single i.p. injection of paclitaxel (2 or 5 mg/kg) to model paclitaxel-induced peripheral neuropathy.

In experiment 2, animals received a single i.p. injection of paclitaxel (5 mg/kg).

In experiment 3, PDTC (100 ng/5μl) was injected i.t. 30 minutes before the injection of paclitaxel (5mg/kg, i.p.).

In experiment 4, RKT (0.1, 0.3, 1 g/kg, p.o.) was administered 1 hour before nociceptive testing once a day for 6 days starting 24 hours before a single injection of paclitaxel (5 mg/kg, i.p.).

In experiment 5, RKT (1 g/kg) was administered 1 hour before nociceptive testing once a day for 5 days starting 24 hours after a single injection of paclitaxel (5mg/kg, i.p.).

In experiment 6, RKT (1 g/kg, p.o.) was administered daily 1 hour before nociceptive testing starting 24 hours after the first of repeated injections of paclitaxel (2 mg/kg, i.p.) on days 1, 3, 5 and 7.

In experiment 7, RKT (1 g/kg, p.o.) and paclitaxel (5 mg/kg, i.p.) were each administered once a day for 3 days. On day 2, [D-Lys^3^]-GHRP-6 (10mg/kg, i.p.) was injected 15 minutes before the administration of RKT.

Finally, in experiment 8, RKT (1 g/kg, p.o.) was administered once a day for 3 days, and paclitaxel (5 mg/kg, i.p.) was injected on day 2, one hour after the administration of RKT.

### Measurement of mechanical hyperalgesia

Mechanical hyperalgesia was determined by probing the plantar surface of the hind paw (von Frey test) with a calibrated plastic filament of a dynamic plantar aesthesiometer purchased from Ugo Basile (Comerio, Italy). Force was applied to the hind paw at a rate of 0.25 g/s; the final force when paw withdrawal observed was measured automatically (mechanical nociceptive threshold). A maximal cut-off of 5 g was used to prevent tissue damage. A significant decrease in the mechanical threshold after the injection of paclitaxel compared with that in vehicle-treated animals was considered to be mechanical hyperalgesia. The mechanical nociceptive threshold was determined as the average of two measurements per mouse.

### Measurement of protein levels of spinal NFκB and pNFκB

Another set of mice was used to determine the protein levels of NFκB and pNFκB in the spinal cord (experiments 2 and 8). In experiment 8, mice were injected with RKT (1 g/kg, p.o.) once a day for 3 days. Paclitaxel (5 mg/kg, i.p.) was injected on day 2 one hour after the administration of RKT. Mice were sacrificed on day 3 one hour after the administration of RKT. The spinal cord was quickly dissected and homogenized with ice-cold RIPA buffer containing 50 mM Tris hydrochloride (pH 7.4), 150 mM sodium chloride, 0.1% sodium dodecyl sulfate, 0.5% sodium deoxycholate, 1% Triton X, 1 mM phenylmethylsulfonyl fluoride, 25 μg/ml leupeptin, 25 μg/ml aprotinin, 10 mM sodium fluoride and 1 mM sodium vanadium oxide. The homogenates were centrifuged at 20,000 ×*g* for 20 min at 4°C, and the protein concentration of the supernatants was measured using a BCA protein assay kit (Thermo Fisher Scientific, Suwannee, GA, USA). Samples were diluted with RIPA buffer to give the same concentration of protein (25 μg/4 μl). Samples were then diluted with an equal volume of 2 × electrophoresis sample buffer containing 2% SDS and 10% glycerol with 0.2M dithiothreitol. Proteins were separated by SDS-PAGE (5–20% gradient gel; Atto, Tokyo, Japan). After electrophoresis, proteins were transferred to a nitrocellulose membrane (Amersham Life Science, Arlington Heights, IL, USA) in Tris/glycine buffer containing 100 mM Tris, 192 mM glycine and 5% methanol. To block non-specific sites, the membranes were soaked in a blocking buffer [3% bovine serum albumin in Tris-buffered saline (pH 7.6) containing 0.1% Tween-20 (TBST)] for 60 min at room temperature. The membranes were immunoblotted overnight at 4°C with rabbit polyclonal antibody against NFκB (1:1000; Cell Signaling Technology, Beverly, MA, USA) and rabbit polyclonal antibody against pNFκB ser 276 (1:1000; Cell Signaling Technology). The membranes were then washed in TBST three times at 10-min intervals and incubated in horseradish peroxidase-conjugated goat anti-rabbit IgG (1:20,000; Cell Signaling Technology) for 90 min at room temperature. After the membranes were washed in TBST five times at 5-min intervals and in Tris-buffered saline (TBS) twice at 5-min intervals, the antigen–antibody peroxidase complex was detected by enhanced chemiluminescence (Thermo Fisher Scientific), and immunoreactive bands were visualized by Light Capture (AE-6981C; Atto). The membranes were washed again in blocking buffer containing 0.1% sodium azide and incubated with antibody against GAPDH (1:100,000; Millipore, Billerica, MA, USA) overnight at 4°C with gentle agitation. Membranes were incubated with second antibody, and image development was performed. The intensity of the band was analyzed and semiquantified by computer-assisted densitometry using a CS analyzer (Atto). Values for NFκB and pNFκB in the spinal cord of mice were normalized by the respective value for GAPDH.

### Statistical analysis

The data are expressed as the mean ± S.E.M. The statistical significance of differences between groups was assessed with Student’s *t*-test (for comparisons of two groups) or one-way or two-way analysis of variance (ANOVA) followed by a post hoc Bonferroni multiple comparisons test (for comparisons among multiple groups). A level of P < 0.05 was considered significant.

## Results

### Paclitaxel induced mechanical hyperalgesia (experiment 1)

**Fig 1:**
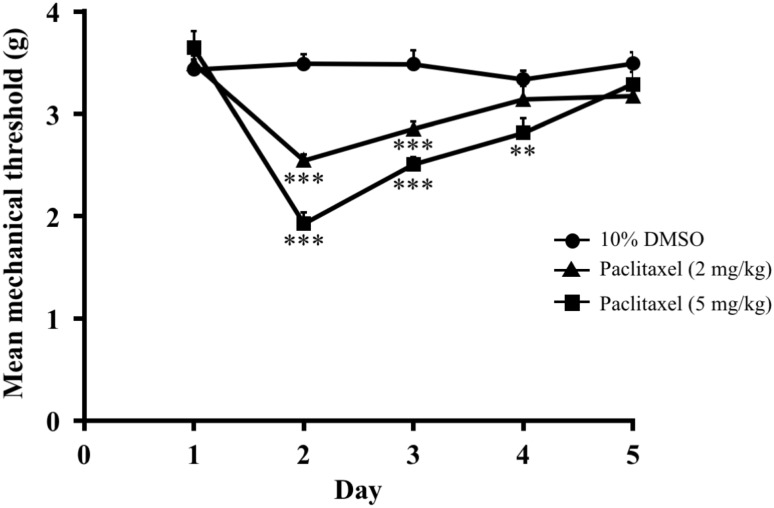
Effect of paclitaxel on mechanical hyperalgesia in mice. Effect of a single administration of paclitaxel (2, 5 mg/kg, i.p.) on mechanical hyperalgesia in mice. The nociceptive threshold was determined by the von Frey filament test. Each point represents the mean ± S.E.M of 8 mice. **P < 0.01, ***P < 0.001 vs. respective 10% DMSO group.

The effect of paclitaxel on mechanical hyperalgesia in mice is shown in Fig 1. A single injection of paclitaxel (2, 5 mg/kg, i.p.) dose-dependently decreased the mechanical nociceptive threshold compared to that in vehicle-treated mice (two-way ANOVA, treatment × time: F_8,84_ = 10.52, P < 0.001). Paclitaxel-treated mice showed a decrease in the mechanical nociceptive threshold that was apparent at 2 days and persisted through 4 days after treatment with paclitaxel.

### Paclitaxel increased protein levels of spinal pNFκB and but not NFκB (experiment 2)

**Fig 2:**
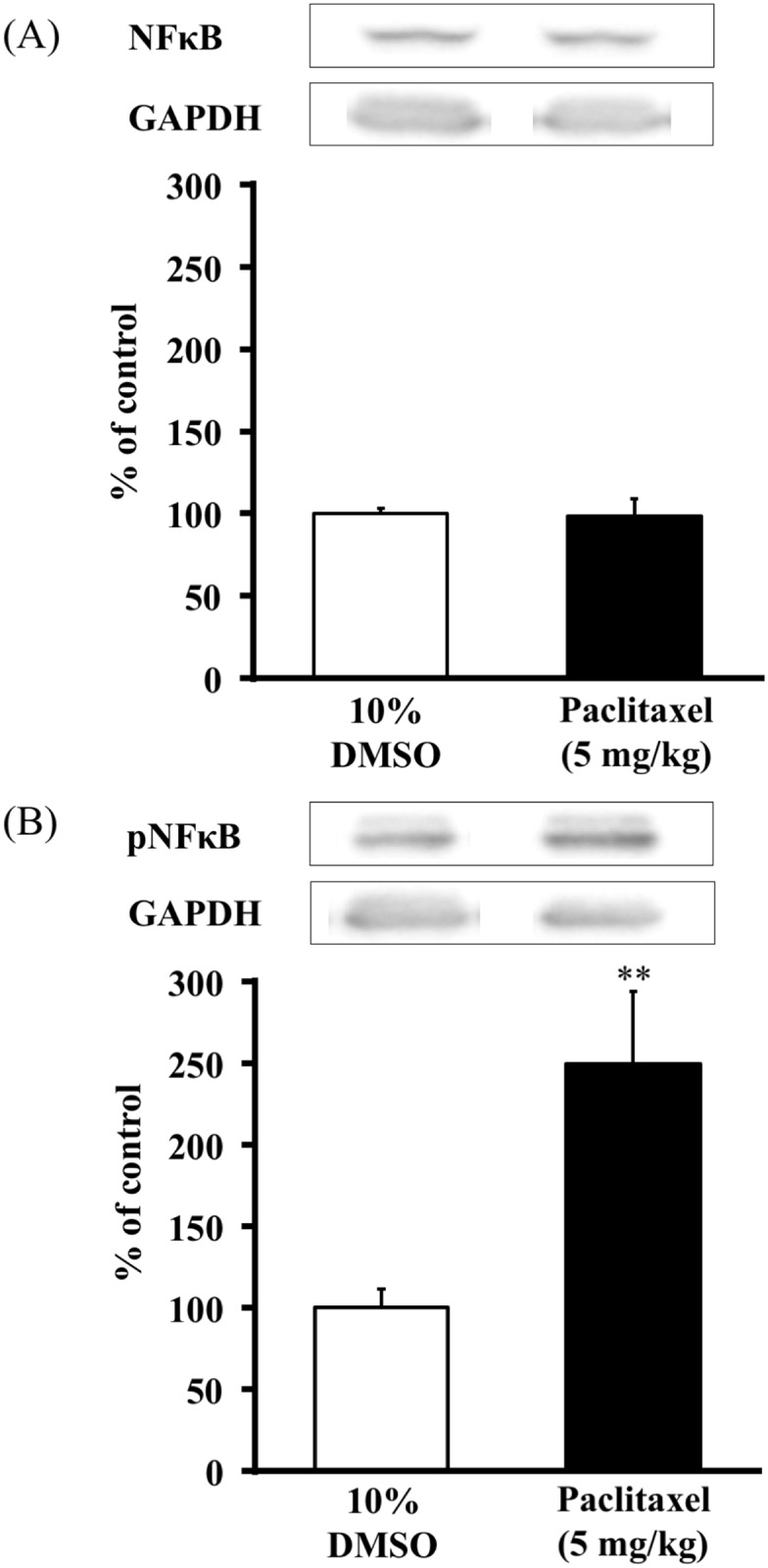
Effect of paclitaxel on the protein levels of spinal NFκB and pNFκB in mice. Immunoblots of (A) NFκB and (B) pNFκB were normalized by GAPDH. The NFκB column represents the mean ± S.E.M of 5–7 mice. The pNFκB column represents the mean ± S.E.M of 3–5 mice. ** P < 0.01 vs. 10%DMSO group.

The effects of paclitaxel on the protein levels of spinal NFκB and pNFκB in mice are shown in Fig 2. The injection of paclitaxel (5 mg/kg, i.p.) did not change the protein levels of spinal NFκB (Fig 2A). However, the injection of paclitaxel (5mg/kg, i.p.) significantly increased the protein levels of spinal pNFκB (Fig 2B).

### NFκB inhibitor, PDTC, suppressed paclitaxel-induced mechanical hyperalgesia (experiment 3)

**Fig 3:**
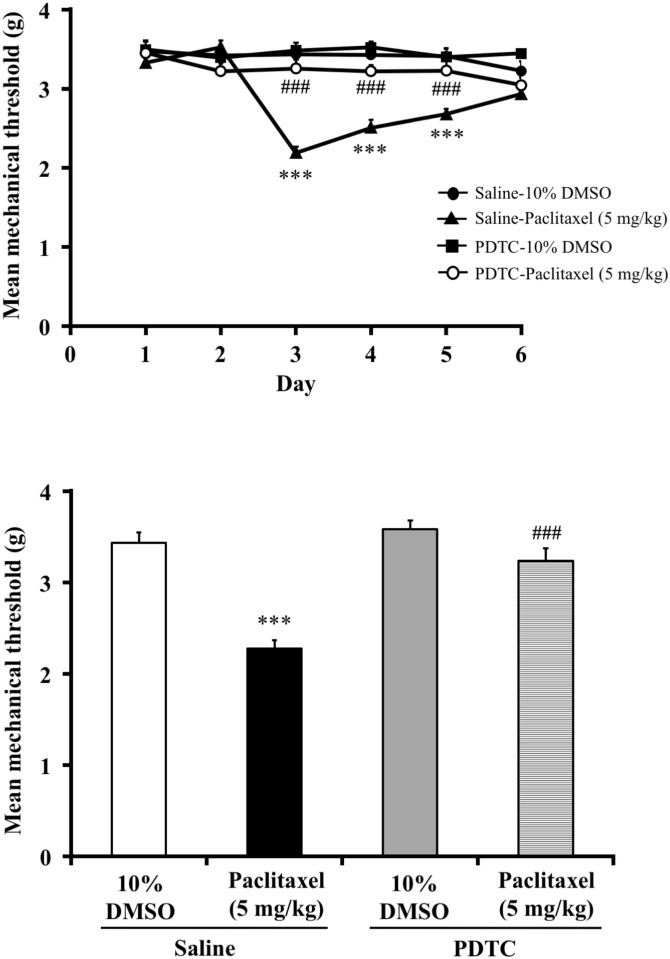
Effect of the NFκB inhibitor, PDTC, on paclitaxel-induced mechanical hyperalgesia in mice. (A) Time course of the effect of PDTC (100 ng/5μl, i.t.) on the mean mechanical threshold in paclitaxel-treated mice. (B) Effect of PDTC (100 ng/5μl, i.t.) on the mean mechanical threshold in paclitaxel-treated mice on day 3. PDTC was administered 30 minutes before the injection of paclitaxel (5 mg/kg, i.p.). The nociceptive threshold was determined by the von Frey filament test. Each point represents the mean ± S.E.M of 8–10 mice. ***P < 0.001 vs. saline-10%DMSO group. ^###^P < 0.001 vs. saline-paclitaxel group.

The effect of PDTC on the mechanical hyperalgesia in paclitaxel (5 mg/kg, i.p.)-treated mice is shown in Fig 3. Pretreatment with PDTC (100 ng/5μl, i.t.) increased the mechanical nociceptive threshold in paclitaxel-treated mice (two-way ANOVA, treatment × time: F_6,75_ = 13.87, P < 0.001; Fig 3A). On day 3, PDTC reversed the decrease in the mechanical nociceptive threshold induced by paclitaxel (one-way ANOVA, treatment: F_3,32_ = 41.97, P < 0.001; Fig 3B).

### Pretreatment with RKT (TJ-43) attenuated paclitaxel (single injection)-induced mechanical hyperalgesia but not post-treatment with RKT (experiments 4 and 5)

**Fig 4:**
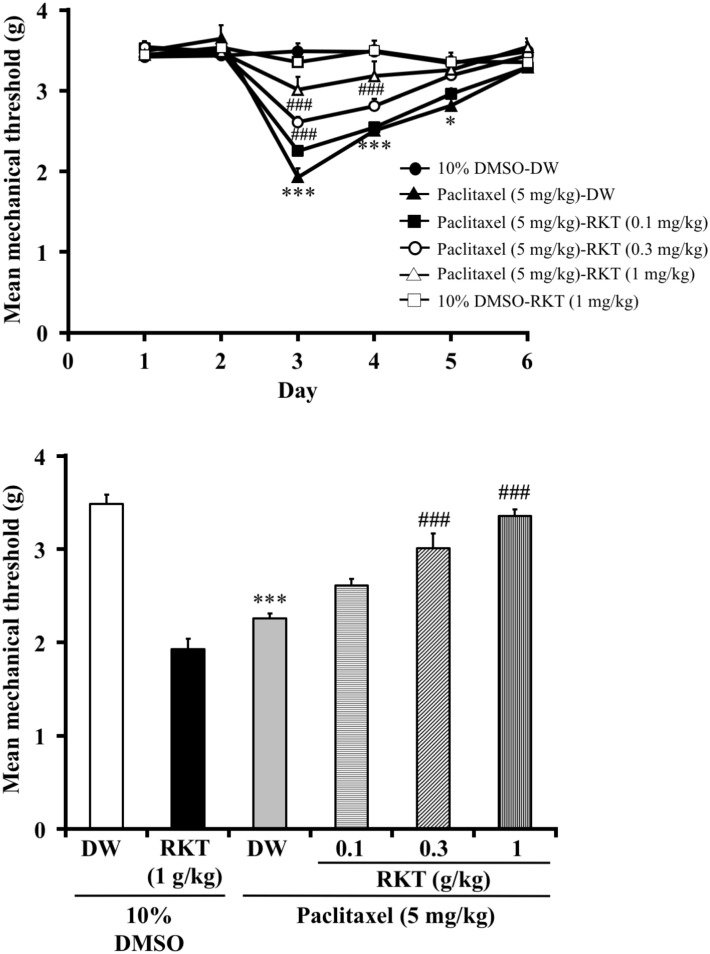
Effect of pretreatment with RKT on paclitaxel (single injection)-induced mechanical hyperalgesia in mice. (A) Time course of the effect of RKT (0.1, 0.3, 1 g/kg p.o.) on the mean mechanical threshold in paclitaxel-treated mice. (B) Effect of RKT (0.1, 0.3, 1 g/kg p.o.) on the mean mechanical threshold in paclitaxel-treated mice on day 3. RKT was administered 24 hours before the injection of paclitaxel (5 mg/kg, i.p.). RKT was administered 1 hour before each nociceptive test. The nociceptive threshold was determined by the von Frey filament test. Each point represents the mean ± S.E.M of 8–9 mice. *P < 0.05, ***P < 0.001 vs. 10%DMSO-DW group. ^###^P < 0.001 vs. paclitaxel-DW group.

**Fig 5:**
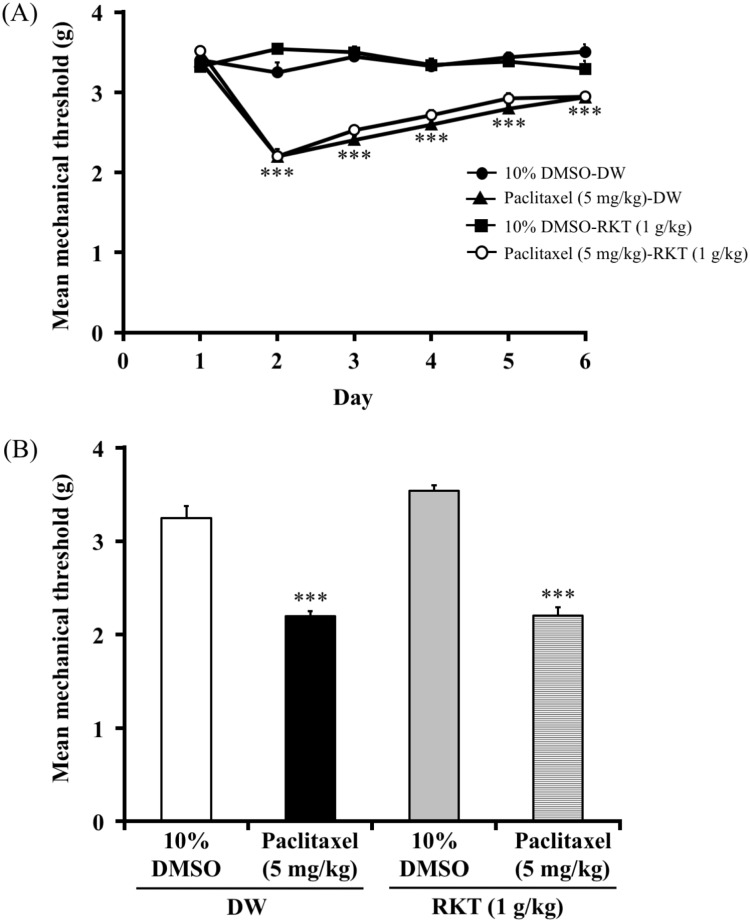
Effect of post-treatment with RKT on paclitaxel (single injection)-induced mechanical hyperalgesia in mice. (A) Time course of the effect of RKT (1 g/kg, p.o.) on the mean mechanical threshold in paclitaxel-treated mice. (B) Effect of RKT (1 g/kg p.o.) on the mean mechanical threshold in paclitaxel-treated mice on day 2. RKT was administered 24 hours after the injection of paclitaxel (5 mg/kg, i.p.). RKT was administered 1 hour before each nociceptive test. The nociceptive threshold was determined by the von Frey filament test. Each point represents the mean ± S.E.M of 7–8 mice. ***P < 0.001 vs. 10%DMSO-DW group.

The effect of RKT on paclitaxel-induced mechanical hyperalgesia in mice is shown in Figs 4 and 5. The administration of RKT (0.1, 0.3, 1 g/kg, p.o.) 24 hours before the single injection of paclitaxel (5mg/kg, i.p.) dose-dependently increased the mechanical nociceptive threshold in mice (two-way ANOVA, treatment × time: F_25,220_ = 7.69, P < 0.001; Fig 4A). On day 3 after the injection of paclitaxel, mice that had been pretreated with RKT showed a reversal of the decrease in the mechanical nociceptive threshold induced by paclitaxel (one-way ANOVA, treatment: F_3,30_ = 19.76, P < 0.001; Fig 4B). However, the administration of RKT (1 g/kg, p.o.) 24 hours after the injection of paclitaxel (5mg/kg, i.p.) did not increase the mechanical nociceptive threshold (two-way ANOVA, treatment × time: F_15,125_ = 12.7, P < 0.001; Fig 5A). On day 2 after the injection of paclitaxel, post-treatment with RKT did not reverse the decrease in the mechanical nociceptive threshold induced by paclitaxel (one-way ANOVA, treatment: F_3,25_ = 70.2, P < 0.001; Fig 5B).

### Pretreatment with RKT (TJ-43) on paclitaxel (repeated injections)-induced mechanical hyperalgesia in mice (experiment 6)

**Fig 6:**
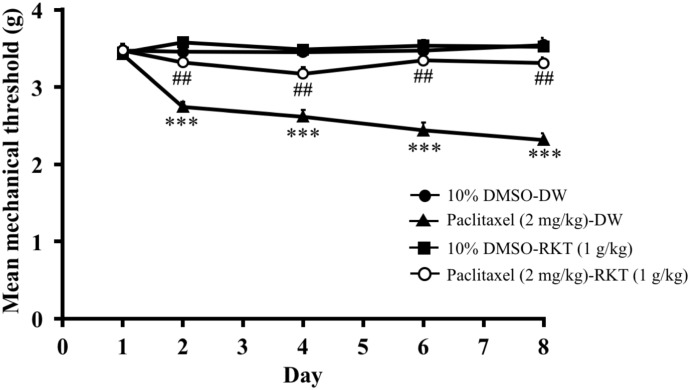
Effect of RKT on paclitaxel (repeated injections)-induced mechanical hyperalgesia in mice. RKT (1g/kg, p.o.) was administered before the injection of paclitaxel (2 mg/kg, i.p.) on days 1, 3, 5 and 7. RKT was administered 1 hour before each nociceptive test. The nociceptive threshold was determined by the von Frey filament test. Each point represents the mean ± S.E.M of 7–8 mice. *** P < 0.001 vs. 10%DMSO-DW group. ^##^P < 0.01 vs. paclitaxel-DW group.

The effect of RKT on paclitaxel (repeated injections)-induced mechanical hyperalgesia in mice is shown in Fig 6. The injection of paclitaxel (5 mg/kg, i.p.) on days 1, 3, 5 and 7 time-dependently decreased the mechanical nociceptive threshold in mice (two-way ANOVA, treatment × time: F_4,48_ = 15.18, P < 0.001). However, repeated administration of RKT (1 g/kg, p.o.) reversed the decrease in the mechanical nociceptive threshold induced by paclitaxel (two-way ANOVA, treatment × time: F_4,52_ = 9.816, P < 0.001).

### Ghrelin receptor antagonist, [D-Lys^3^]-GHRP-6, suppressed antihyperalgesic effect of RKT (TJ-43) against paclitaxel-induced hyperalgesia in mice (experiment 7)

**Fig 7:**
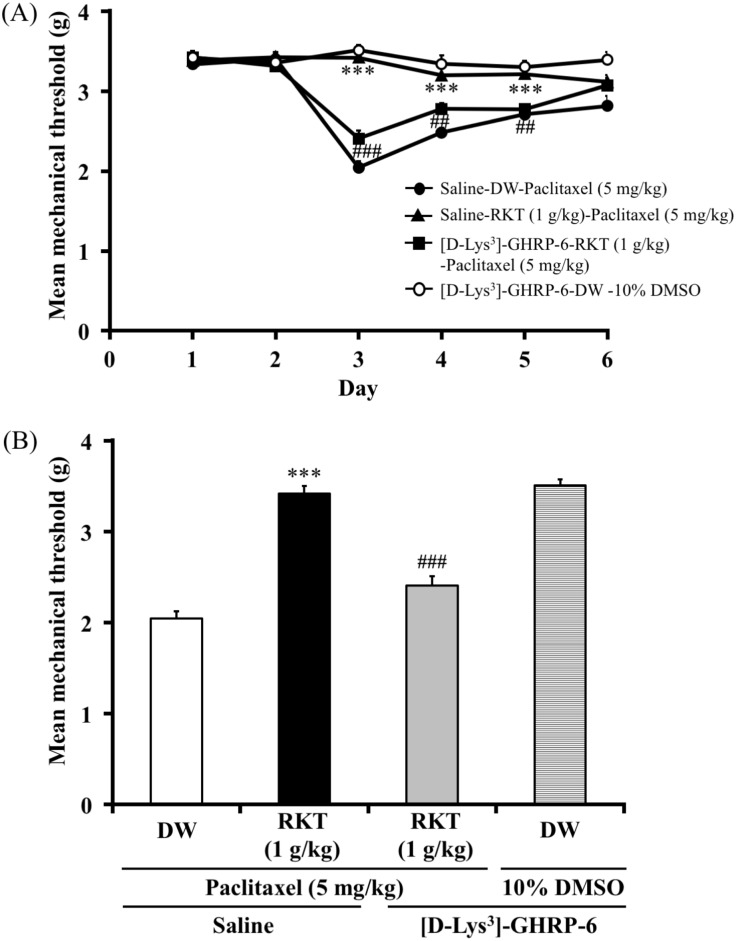
Effect of [D-Lys^3^]-GHRP-6 on the antihyperalgesic effect of RKT against paclitaxel-induced mechanical hyperalgesia in mice. (A) Time course of the effect of [D-Lys^3^]-GHRP-6 (10 mg/kg, i.p.) on the antihyperalgesic effect of RKT (1 g/kg, p.o.) on the mean mechanical threshold in paclitaxel-treated mice. (B) Effect of [D-Lys^3^]-GHRP-6 (10 mg/kg, i.p.) on the antihyperalgesic effect of RKT (1 g/kg, p.o.) on the mean mechanical threshold in paclitaxel-treated mice on day 3. Paclitaxel (5 mg/kg, i.p.) and RKT (1 g/kg, p.o.) were injected once a day for 3 days. [D-Lys^3^]-GHRP-6 (10mg/kg, i.p.) was injected on day 2, 15 min before the administration of RKT. RKT was administered 1 hour before each nociceptive test. The nociceptive threshold was determined by the von Frey filament test. Each point represents the mean ± S.E.M of 6–8 mice. ***P < 0.001 vs saline-DW-paclitaxel group, ^###^P < 0.001 vs saline-RKT-paclitaxel group.

The effect of [D-Lys^3^]-GHRP-6 on the antihyperalgesic effect of RKT against paclitaxel-induced neuropathic pain is shown in Fig 7. Pretreatment with [D-Lys^3^]-GHRP-6 (10mg/kg, i.p.) decreased the RKT (1 g/kg, p.o.)-induced increase in the mechanical nociceptive threshold in paclitaxel (5 mg/kg, i.p.)-treated mice (two-way ANOVA, treatment × time: F_8,76_ = 17.71, P < 0.001; Fig 7A). On day 3, [D-Lys^3^]-GHRP-6 reversed the antihyperalgesic effect of RKT against paclitaxel-induced hyperalgesia in mice (one-way ANOVA, treatment: F_3,24_ = 74.6, P < 0.001; Fig 7B).

### RKT (TJ-43) decreased the protein levels of spinal pNFκB but not NFκB by paclitaxel (experiment 8)

**Fig 8:**
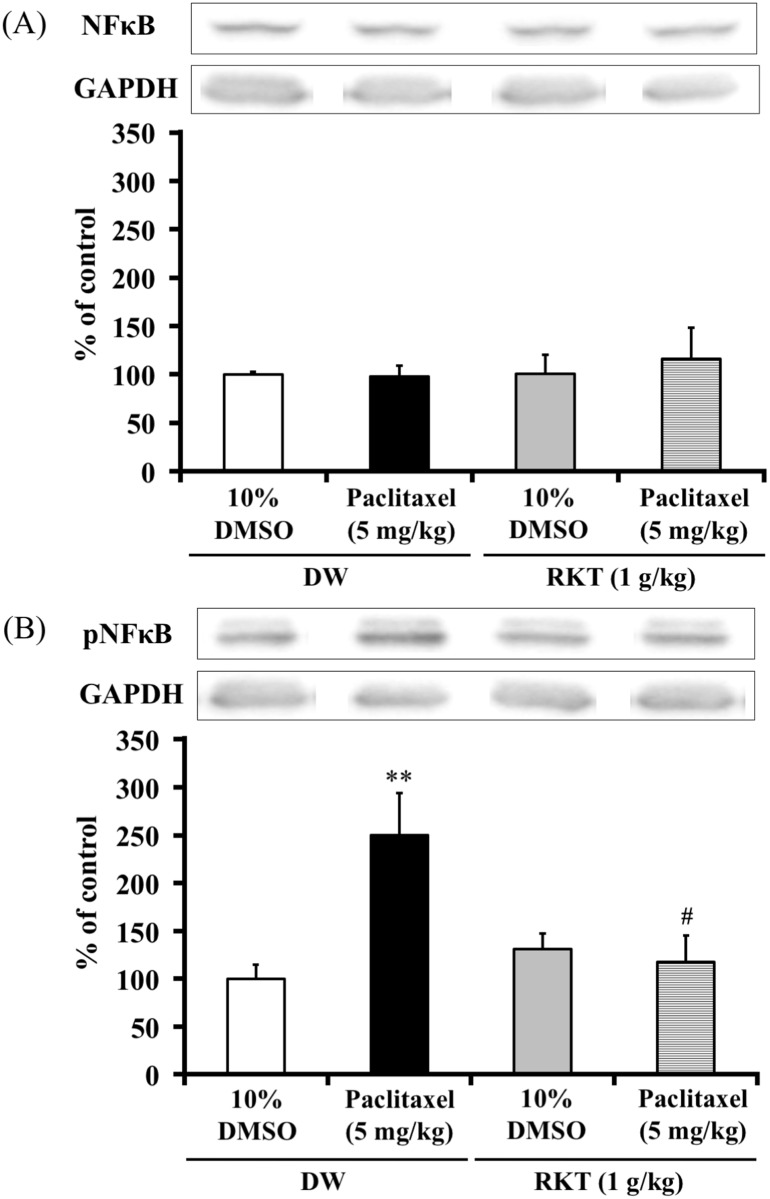
Effect of RKT on the protein levels of spinal NFκB and pNFκB in mice treated with paclitaxel. Immunoblots of (A) NFκB and (B) pNFκB were normalized by GAPDH. The NFκB column represents the mean ± S.E.M of 5–7 mice. The pNFκB column represents the mean ± S.E.M of 3–5 mice. **P < 0.01 vs. 10%DMSO-DW group. ^#^P < 0.05 vs. paclitaxel-DW group.

The effects of pretreatment with RKT on the protein levels of spinal NFκB and pNFκB in mice treated with paclitaxel are shown in Fig 8. Pretreatment with RKT (1 g/kg, p.o.) did not change the protein levels of spinal NFκB following treatment with paclitaxel (5 mg/kg, i.p., Fig 8A). However, pretreatment with RKT (1 g/kg, p.o.) reversed the increase in the protein levels of spinal pNFκB following treatment with paclitaxel (5 mg/kg, i.p., Fig 8B).

## Discussion

Our results demonstrated that a single injection of paclitaxel dose-dependently induced mechanical hyperalgesia in mice. CIPN is a common toxicity associated with multiple chemotherapeutic agents, including the vinca alkaloid vincristine, the taxane paclitaxel, and the platinum-based drug oxaliplatin, the microtubule-targeting agent pemetrexed, the proteasome inhibitor bortezomib, and angiogenesis inhibitors. The reported incidence of CIPN ranges between 30% and 40% [20]. CIPN is characterized by burning pain, numbness, and tingling in the hands and feet [21–24]. CIPN can be painful and significantly degrade the quality of life.

Paclitaxel is a microtubule-binding antineoplastic drug. It is one of the most widely used chemotherapeutic agents for various types of solid tumor such as breast, ovarian, and non-small-cell lung cancer [1]. However, treatment with paclitaxel is associated with peripheral neuropathy as a major side effect. Several animal models have been used to examine the effects of paclitaxel. Injection of a low dose of paclitaxel induces mechanical hyperalgesia and allodynia [25–27]. Furthermore, paclitaxel increases the mRNA expression of IL-1β and tumor necrosis factor-α (TNF-α), and immune cell markers in lumbar DRG [28].

Our current study showed that paclitaxel increased the protein levels of spinal pNFκB, but not those of spinal NFκB. NFκB is a pleiotropic transcriptional factor that is sequestered by binding to IκB. After activation, IκB is phosphorylated by IKKs, and the nuclear factor is then translocated into the nucleus [29]. Moreover, NFκB regulates the gene expression of many factors associated with pain, such as TNF-α and IL-6 [30, 31]. The activation of NFκB occurs in the DRG and spinal cord, and plays a role in nociceptive information. Increased NFκB activation in the lumbar DRG has been observed after partial sciatic nerve injury in rat [32]. Interestingly, NFκB and its downstream pro-inflammatory cytokines, such as TNF-α, IL-6, and IL-1β, also play important roles in neuropathic pain [33]. Intrathecal administration of the NFκB inhibitor PDTC attenuates mechanical allodynia in neuropathic pain [34]. Several studies have reported that paclitaxel induces NFκB activation in several human cancer cells [35, 36].

The present study demonstrated that pretreatment with the NFκB inhibitor, PDTC, attenuated paclitaxel-induced mechanical hyperalgesia. Our results may contribute to the treatment of paclitaxel-induced hyperalgesia via activation of the NFκB pathway.

In the present study, we demonstrated that pretreatment with RKT dose-dependently attenuated paclitaxel-induced mechanical hyperalgesia. However, post-treatment with RKT did not attenuate paclitaxel-induced mechanical hyperalgesia. These results suggest that it may be possible to use RKT preventively to produce an antihyperalgesic effect against paclitaxel-induced neuropathic pain. RKT is a Kampo (Japanese herbal) medicine that has been prescribed to patients in Japan with various upper gastrointestinal symptoms, including GERD, NERD and functional dyspepsia, for more than 20 years [37, 38]. For the first time, our results showed that RKT had an antihyperalgesic effect against paclitaxel-induced neuropathic pain.

The present result showed that repeated injection of paclitaxel time-dependently induced mechanical hyperalgesia. Paclitaxel-induced neuropathic pain is characterized by hypersensitivity that develops early after the beginning of treatment and may persist for weeks or even years after the termination of therapy. Clinical evidence has shown that paclitaxel-induced neuropathic pain depends on several factors, such as the number of doses per cycle, the treatment schedule, the duration of infusion and the cumulative dose [39–44]. The risk of paclitaxel-induced neuropathic pain is proportional to the dose of paclitaxel administered. Grade 3 or 4 sensory neuropathic pain arises in 20–35% of patients with a dose of 250 mg/m^2^, but in only 5–12% with doses ≤ 200 mg/m^2^ [45]. This result suggests that the repeated injection of paclitaxel in this animal model may mimic the paclitaxel-induced neuropathic pain observed in clinical practice.

To examine whether RKT is effective for attenuating paclitaxel-induced neuropathic mechanical hyperalgesia, we administered RKT to mice that were repeatedly injected with paclitaxel (days 1, 3, 5, and 7). As a result, repeated pretreatment with RKT attenuated paclitaxel-induced mechanical hyperalgesia. These results suggest that chronic administration of RKT is effective for treating paclitaxel-induced neuropathic pain. Recently, it has been reported that RKT increased the level of ghrelin, a gastrointestinal hormone, in humans and normal mice [46]. RKT is consisted eight components (*Aurantii* pericarpium, *Ginseng* radix, *Zingiberis* rhizoma, *Jujubae* (*Zizyphi*) frucutus, *Pinellia* tuber, *Atractylodis* rhizoma, *Glycyrrhiza* radix and *Poria cocos* (Hoelen)). It has been reported that RKT increased the ghrelin level. Especially, hesperidin from *Aurantii* pericarpium suppressed the cisplatin-induced decrease in the plasma ghrelin level and increases food intake [47]. Ghrelin is an endogenous ligand for GHSR-1a (i.e., ghrelin receptor), and has been shown to exert various effects under both physiological and pathological conditions. Ghrelin showed anti-inflammatory activity in a murine model by inhibiting levels of TNF-α, IL-1β and IL-6. Administration of ghrelin significantly reduces serum levels of cytokines such as TNF-α, IL-1β and IL-6, after lipopolysaccharide (LPS) challenge [48].

In the present study, we suggested that pretreatment with the ghrelin receptor antagonist, [D-Lys^3^]-GHRP-6, reversed the RKT-induced attenuation of paclitaxel-induced mechanical hyperalgesia. Several studies have reported that ghrelin plays a role in the control of pain. Ghrelin has been shown to have antinociceptive effects in several experimental models [49–55]. It has been reported that ghrelin inhibits the development of hyperalgesia induced by the injection of carrageenan rats [53], and GHSR-1a is involved in the anti-inflammatory activity of ghrelin [54]. It has also been reported that ghrelin attenuates pain behaviors by decreasing TNF-α and IL-1β levels in the spinal cord and in a sciatic nerve injury model of neuropathic pain.

In agreement with our results regard the antihyperalgesic effect of RKT against paclitaxel-induced neuropathic pain, we demonstrated that pretreatment with RKT inhibited the paclitaxel-induced increase in the protein levels of spinal pNFκB. On the other hand, treatment with paclitaxel did not change the protein levels of spinal NFκB. Several studies have reported that ghrelin prevents the activation of NFκB in humans [56]. These results that RKT may inhibit the phosphorylation of spinal NFκB by increasing ghrelin activity. In this study, we found that RKT exerts an antihyperalgesic effect in paclitaxel-induced neuropathic pain by suppressing the activation of spinal NFκB.

At present, there are several new approaches to the treatment of neuropathic pain, such as antidepressants (tricyclic antidepressants, SNRIs), gabapentin, cyclooxygenase (COX) inhibitors, antioxidants, mitochondrial protective agents, reverse paclitaxel-induced mechanical hypersensitivity and cold allodynia [57–62]. Paclitaxel-treated animals show the attenuation of peripheral neuropathy by the anti-inflammatory thalidomide, minocycline or the anti-inflammatory cytokine IL10 [63, 64]. These results suggest RKT might be a potential new therapeutic agent for the treatment of paclitaxel-induced neuropathic pain.

We proposed that paclitaxel was activated spinal NFκB. Also, pretreatment with RKT attenuated paclitaxel-induced mechanical hyperalgesia but not by post-treatment with RKT. Based on these results, we proposed that RKT suppressed paclitaxel-induced activation of NFκB, but not after activated NFκB. Therefore, we examined the effect of RKT on the NFκB levels after acute treatment with paclitaxel. Farther, RKT inhibits the cisplatin-induced decrease in the ghrelin level and increases food intake [47]. RKT administered in combination with an anti-emetic drug proved useful against the anorexia and vomiting as adverse reactions to chemotherapy. Therefore, we investigated the mechanism by which co-administration of RKT with anticancer agents proves useful in clinical practice by attempting to show that RKT prevents not only the decreases of appetite but also the hyperalgesic in paclitaxel-induced neuropathic pain.
